# Violence and harassment against healthcare workers: a psychological-clinical perspective on a survey in a Policlinic Hospital

**DOI:** 10.3389/fpubh.2025.1607011

**Published:** 2025-07-22

**Authors:** Ermanno Vitale, Francesca Vella, Serena Matera, Silvia Mazzeo, Chiara Farrauto, Veronica Filetti, Venerando Rapisarda, Giorgia Farruggio, Noemi Maria Vitale, Eleonora Uccelli, Abdulnaser Fakhrou, Lucia Rapisarda, Giorgio Bertolazzi, Davide Matera, Pasquale Caponnetto

**Affiliations:** ^1^Department of Medicine and Surgery, University of Enna “Kore”, Enna, Italy; ^2^Department of Clinical and Experimental Medicine, University of Catania, Catania, Italy; ^3^Department of Education Sciences, Section of Psychology, University of Catania, Catania, Italy; ^4^Center of Excellence for the Acceleration of Harm Reduction (CoEHAR), University of Catania, Catania, Italy; ^5^Psychological Sciences, College of Education, Qatar University, Doha, Qatar; ^6^Spinal Unit AOE “Cannizzaro”, Catania, Italy; ^7^Mediterranean Institute of Oncology (IOM), Viagrande, Italy

**Keywords:** workplace violence, healthcare workers, occupational health, mental health, post-traumatic stress

## Abstract

**Objective:**

The aim of this study was to assess the prevalence and psychological impact of workplace violence (WPV) among healthcare workers (HCWs) in a large university hospital in Southern Italy, and to identify occupational and demographic risk factors.

**Methods:**

A cross-sectional observational study was conducted from January to December 2023. A total of 997 HCWs voluntarily completed a validated, anonymous questionnaire based on the “Workplace Violence in the Health Sector” (WVHS) tool. The instrument assessed exposure to different types of WPV (physical, verbal, bullying, sexual and racial harassment) and its emotional and occupational consequences. Statistical analyses included descriptive statistics, linear regression and ANOVA (*p* < 0.05).

**Results:**

27% of HCWs reported experiencing at least one form of WPV in the past 12 months. Verbal violence was the most common (85%), followed by bullying (26%). Nurses, resident doctors, younger workers, and those working night shifts were more frequently affected. Victims showed higher use of psychotropic drugs and psychotherapy (*p* < 0.001).

**Conclusion:**

WPV is highly prevalent and underreported in healthcare settings, with significant mental health repercussions. The findings highlight the need for preventive strategies, institutional support, and the key role of occupational physicians in early identification and intervention.

## Introduction

Workplace violence (WPV) has become a serious and multidimensional problem that negatively affects the professional and personal lives of workers (1). The World Health Organization (WHO) defines WPV as *“incidents in which staff are abused, threatened or assaulted in circumstances related to their work, including commuting to and from work, that involve an implicit or explicit threat to their safety, well-being or health”* (2). The definition includes two broad categories of violence: physical and non-physical abuse, which in turn includes verbal abuse, bullying, harassment, or threats (3, 4).

The highest rate of exposure to WPV occurs in health care institutions (4–6); in particular, healthcare workers (HCWs) are four times more likely to experience WPV than workers in other occupational sectors (3, 7, 8). It is thought that this high vulnerability of HCWs is mainly related to the complex relationships that HCWs have to manage with patients and their relatives (9), who may sometimes feel frustrated due to certain aspects related to the organization of the healthcare facility and other factors, such as long waiting times or an unfavorable prognosis, thus becoming aggressive (10). Furthermore, certain organizational factors such as the number of patients, social support or an unsafe working environment have been shown to be associated with an increased risk of WPV in the healthcare sector (11). A review by Kumari et al. (12) highlighted a number of factors that could initiate episodes of violence in this context, classified as professional, patient-related, organizational and social.

In addition, HCWs may also suffer violence from their colleagues. This type of violence is known as *“lateral violence”* or *“horizontal violence*,” and can occur between HCWs at all levels, from newly recruited staff to managers (13).

Many studies have investigated WPV in different countries; a meta-analysis by Liu et al. (14), which collected studies involved on all continents, showed that 61.9% of the participants had been exposed to any form of violence at work and 24.4% had experienced physical violence in the previous year.

In 2022, a cross-sectional study conducted on HCWs operating in several Italian Regions (15) with the aim of investigating the prevalence of some forms of WPV showed that the female gender was significantly more affected (16.4%) and the most affected age group was 35 to 39 years old. A significant difference was found between the different regions, such that those in the North had a higher incidence of bullying (17%).

In some countries, the phenomenon of WPV against HCWs might be more widespread than the literature shows. Several factors could be behind this, such as inadequate reporting mechanisms or a lack of trust in the legal system and the fear of stigmatizing those who denounce violence (16).

Furthermore, HCWs might be reluctant to report violence for fear of retaliation or negative consequences for their career (17). In addition to this, HCWs might also believe that violence is a normal part of their work and that complaints will never be taken seriously; in fact, studies show that the phenomenon of WPV is seen and accepted by HCWs as a normal part of work in care settings where it occurs most frequently, such as emergency rooms and psychiatric wards, and, consequently, is rarely reported (18).

The aim of the study is to investigate cases of violence among HCWs working in one of the biggest Policlinic Hospital of South Italy, to estimate the prevalence of this phenomenon, hypothesize the psychological-clinical impact on their lives and plan possible prevention and intervention strategies.

## Subjects and methods

### Study design

The study was designed as a cross-sectional observational survey, aimed at assessing the prevalence of work-related violence against HCWs in the Policlinic Hospital of University of Catania (Sicily Island, Italy). Data were collected through an anonymous self-administered questionnaire, with the aim of identifying factors associated with violence and its psychological impact on operators.

### Participants

The participants were HCWs operating in the Policlinic Hospital of University of Catania. Participation in the study took place on a voluntary basis during the mandatory health surveillance activities, according to Italian Law Decree (DL) 81/08, from January 2023 to December 2023. All the workers invited to take part in the project were informed about the objectives and procedures of the study. Informed consent was obtained from all subjects involved in the study. It was not necessary to receive confirmation from the Ethical Committee as the activity is governed by the Law Decree (DL) 81/08. In particular, under article 25 of the DL 81/08, the occupational physician must collaborate with the employer in the risk assessment and can also propose voluntary health promotion programs in the workplace.

Inclusion criteria: work as HCWs at Policlinic Hospital for at least 12 months. Exclusion criteria: not completing the questionnaire; not signing the informed consent; being workers of Policlinic Hospital for less than 12 months.

### Instruments and procedures

In the study was used an adaptation of the Italian version of the questionnaire *“Workplace Violence in the health sector”* (WVHS), which allows to identify and assess the incidence of violence levels among HCWs (19). This version has been previously translated, culturally adapted, and psychometrically validated in the Italian context. In particular, La Torre et al. (15) reported its internal consistency and structural reliability in a large cross-sectional study involving Italian healthcare workers. Based on the availability of this validated version and to ensure methodological consistency with national literature, no further validation was performed within our sample. Overall, the questionnaire measured the emotional impact and occupational distress experienced following an event of violence in the workplace, and was structured in six sections: (1) Collection of personal data; (2) Section on the frequency of incidents of physical violence in the last 12 months in the workplace; (3) Section on the frequency of incidents of verbal violence in the last 12 months in the workplace; (4) Section on the frequency of bullying incidents in the workplace in the last 12 months; (5) Section on the frequency of incidents of sexual harassment in the last 12 months in the workplace; (6) Section on the frequency of racial harassment incidents in the last 12 months in the workplace.

Each of the sections on incidents of violence was divided into a series of questions aimed at investigating not only the presence of that specific form of violence and its frequency, but also the perpetrator of the violence, the reactions of the victim, whether any action was taken to investigate the cause, whether the incident was reported or not, and if not why.

During the study period each HCWs was offered to voluntarily participate in the study. In particular, by means of a QRcode, each operator had access to the anonymous WVHS questionnaire. The use of a QRcode enabled participants to directly access the questionnaire via their personal or institutional mobile devices, ensuring rapid and user-friendly access without the need for physical contact or login credentials. The survey was hosted on the *Google Forms platform*, chosen for its cross-platform compatibility and stable performance in healthcare environments.

### Statistical analysis

Subsequently, the collected data were statistically analyzed to gain an in-depth understanding of the phenomenon. First, descriptive analyses were conducted to examine the distribution of questionnaire responses and to calculate the descriptive statistics for each variable. Next, a logistic regression model was adapted to identify the variables significantly associated with violence events involving healthcare workers (HCWs). The variables included in the regression model were supervised selected, with highly correlated variables excluded to avoid multicollinearity. We decided to exclude the worker’s age from the regression model because it highly correlates with years of service (Chi-squared test *p*-value < 2.2e−16). To prevent redundancies in the regression model, we calculated the variance inflation factors (VIF) for all predictors using the vif function from the R package “car.” This analysis confirmed the absence of multicollinearity in the model. Finally, the goodness of fit of the logistic regression model was evaluated using the Hosmer-Lemeshow test (*p*-value = 0.83), confirming a good fit for the model.

The variables were considered statistically significant when *p* < 0.05. The statistical analysis was carried out using the R software (4.5.0).

## Results

**Figure 1:**
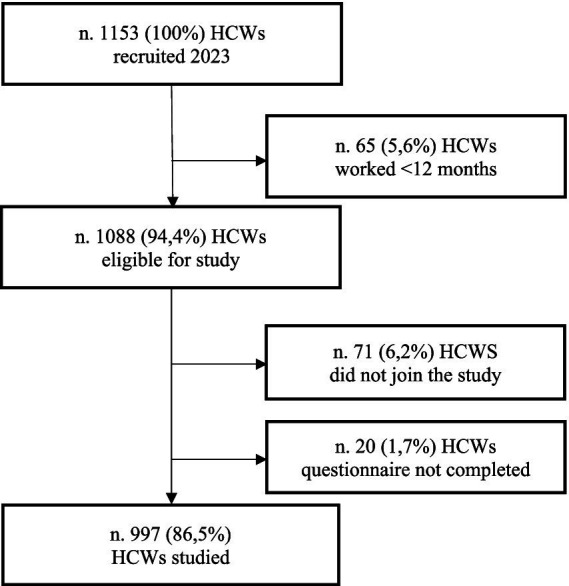
Flowchart for recruiting HCWs in the period from January 2023 to December 2023.

Of a total of 1,153 (100%) HCWs who presented for a visit during the study period (year 2023), 65 (0.6%) HCWs were not admitted because they had been working for less than 12 months, many in fact had presented for the mandatory hiring visit by law. Other 91 (7.9%) HCWs did not take part in the study: 71 (6.2%) decided to not join to the study and the remaining 20 (1.7%) did not complete correctly the questionnaire (see Figure 1).

Therefore, 997 (100%) HCWs correctly completed the questionnaire and were admitted to the study. Of the 997 (100%), 57% (*n* = 568) were women, mean age 38.2 ± 3.7 years and 43% (*n* = 429) were men, mean age 39.7 ± 4.2 years. The sample was equally distributed among: medical doctor (24%); resident doctor (24%); nurses (27%) and healthcare auxiliary (25%). Table 1 shows the main characteristics of the sample. The sample consisted of subjects of Italian nationality.

**Table 1:** Sample characteristics.

Sample *n* = 997 (100%)	Values
Gender (*n*, %)	Male 429 (43%)
	Female 568 (57%)
Age (years, mean ± SD)	39.5 ± 12.6
Job role (*n*, %)	Medical Doctor 243 (24%)
	Resident doctor 242 (24%)
	Nurse 261 (27%)
	Healthcare auxiliary 249 (25%)
Years of service (mean ± SD)	20.1 ± 13.9
Night job (*n*, %)	653 (65%)
Smokers (*n*, %)	313 (31%)
Pack-years (mean ± SD)	19.2 ± 7.3
Alcohol consumption ≥1 drink/week (*n,* %)	184 (18%)
Use of psychotropic drugs (*n*, %)	14 (1.4%)
Psychotherapy (*n*, %)	32 (3.2%)

The length of service was approximately 20 years, 65% (*n* = 653) of the HCWs performed shift work including night work. 31% (*n* = 313) were smokers, on average approximately 19 packs of cigarettes/year; 18% (*n* = 184) regularly consumed alcoholic beverages. In 14 (1.4%) cases the HCWs regularly took psychiatric drugs and in 32 (3.2%) cases they were regularly followed by a psychotherapist.

73% (*n* = 730) of the participants stated that they had never been victims of violence at work; instead, 27% (*n* = 267) of the HCWs stated that they had experienced one or more forms of violence at work in the previous 12 months. Table 2 reports the main characteristics of HCWs divided according to whether they experienced violence at work or not.

**Table 2:** Sample characteristics of HCWs divided according to whether they experienced violence at work or not.

	HCWs no violence victim	HCWs violence victim
HCWs (*n*, %)	730 (100%)	267 (100%)
Gender (*n*, %)	Male 311 (43%)	Male 118 (44%)
	Female 419 (57%)	Female 149 (56%)
Age (years, mean ± SD)	39.3 ± 14.2	38.9 ± 11.5
20–29	213 (29%)	84 (31.5%)
30–39	224 (31%)	84 (31.5%)
40–49	128 (18%)	41 (15%)
50–59	124 (17%)	48 (18%)
60–69	41 (5%)	10 (4%)
Job role (*n*, %)	Medical Doctor 176 (24%)	Medical Doctor 67 (25%)
	Resident doctor 177 (24%)	Resident doctor 65 (24%)
	Nurse 183 (25%)	Nurse 78 (30%)
	Healthcare auxiliary 192 (27%)	Healthcare auxiliary 57 (21%)
Years of service (mean ± SD)	22.4 ± 17.6	20.4 ± 11.2
Night job (*n*, %)	466 (64%)	187 (70%)
Smokers (*n*, %)	220 (30%)	93 (34%)
Pack-years (mean ± SD)	19.1 ± 4.3	19.5 ± 8.4
Alcohol consumption ≥ 1 drink/week (*n*, %)	141 (19%)	43 (18%)
Use of psychotropic drugs (*n*, %)	1 (0.1%)	13 (4.9%)
Psychotherapy (*n*, %)	2 (0.3%)	31 (11.6%)

Of the total 267 (100%) subjects who had experienced violence, the majority belonged to the two lowest age ranges: 20–29 age group (*n* = 84, 31.5%) and 30–39 age group (*n* = 84, 31.5%); the remaining part was equally distributed in the age groups between 40 and 49 (*n* = 41, 15%) and 50–59 (*n* = 48, 18%) years. A very low percentage (*n* = 10, 4%) was recorded in the older age group.

The differences with respect to gender are not excessively marked, but there is still a higher frequency in women, with a percentage of 56% (*n* = 149), while in men there is a percentage of 44% (*n* = 118).

As far as job role is concerned, the group of nurses seems to be the most affected, recording a percentage of 30% (*n* = 78), followed by doctors with 25% (*n* = 67), by resident doctors with 24% (*n* = 65), then the group of HCWs working as healthcare auxiliary with 21% (*n* = 57). 58% (*n* = 155) of HCWs performed night work regularly.

Of these 267 (100%) HCWs victims of violence, 22% (*n* = 53) had experienced two different forms of violence, 4% (*n* = 12) had experienced three, and 3% (*n* = 7) had experienced four different forms of violence in the last 12 months.

**Figure 2:**
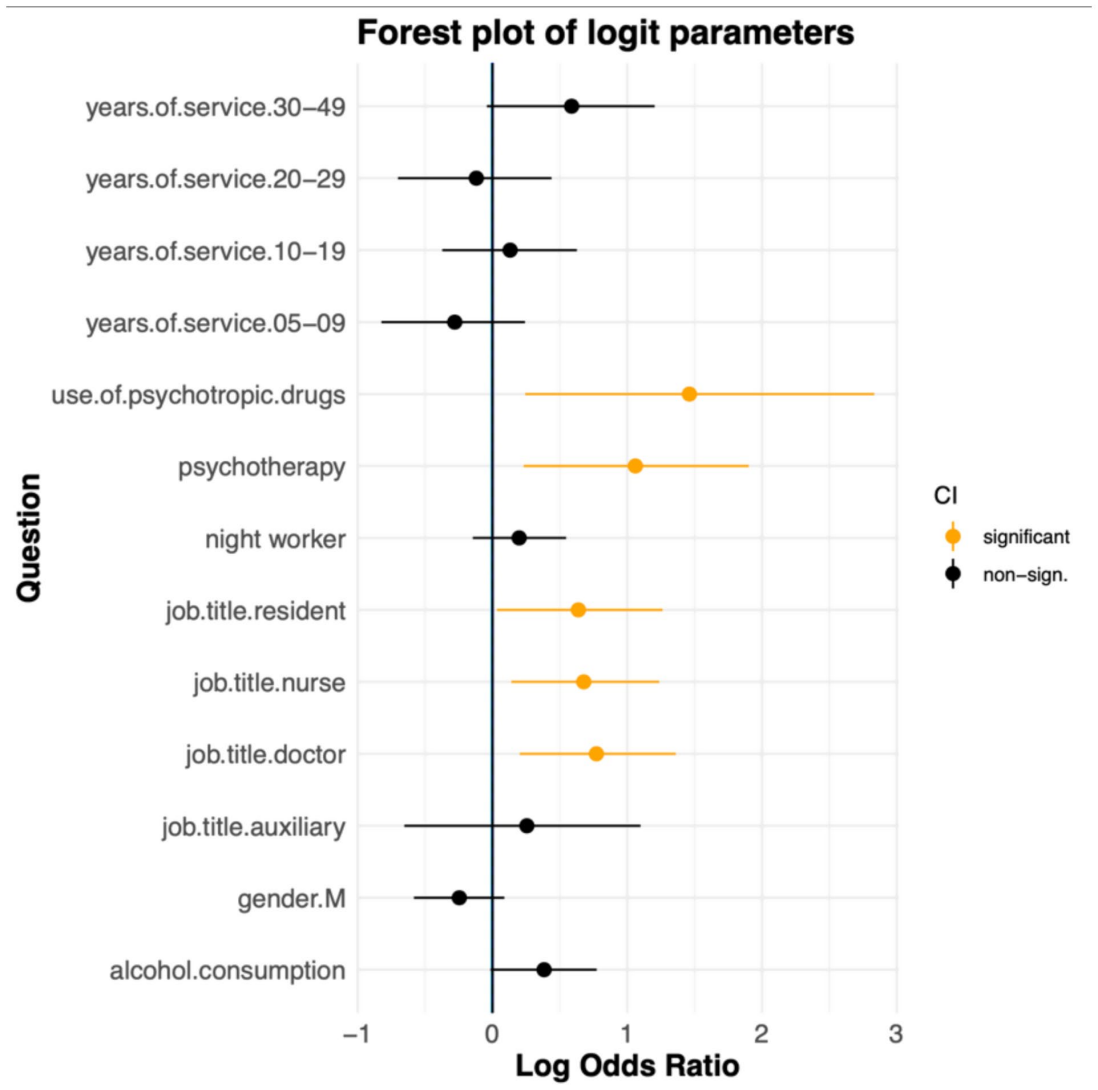
The forest plot shows the estimated log odds ratios from a multiple logistic regression model examining factors associated with experiencing workplace violence. Each point represents the log-OR for a given variable, while the horizontal lines show the corresponding 95% confidence intervals. Variables with statistically significant associations (*p*-value < 0.05) are highlighted in orange. The vertical line at zero indicates no association between the variable and the outcome.

To identify the variables significantly associated with violence events, we adapted a multiple logistic regression model considering the presence of violence as the outcome variable (Table 3). A significant use of psychotropic drugs and psychotherapy sessions is observed. Furthermore, the categories significantly more at risk were doctors, nurses, and resident doctors. The Forest plot of logistic parameters (log-ORs) highlights the significant variables (see Figure 2).

**Table 3:** Results of the logistic regression model evaluating the association between the selected explanatory variables and the outcome.

Variable	Coefficient	Std. Error	ci.lower	ci.upper	OR	*p*-value
Intercept	−1.79	0.27	−2.35	−1.26		
Gender (baseline: female)						
Male	−0.24	0.17	−0.58	0.09	0.78	0.15
Years of service (baseline: years < 5)						
05–09	−0.28	0.27	−0.82	0.24	0.75	0.3
10–19	0.13	0.25	−0.37	0.62	1.14	0.61
20–29	−0.12	0.29	−0.7	0.44	0.89	0.68
30–49	0.58	0.31	−0.04	1.2	1.79	0.064
Job title (baseline: other hospital stuff)						
Auxiliary	0.25	0.44	−0.65	1.09	1.29	0.56
Nurse	0.67	0.28	0.14	1.24	1.97	0.015*
Doctor	0.77	0.29	0.2	1.36	2.16	0.0089**
Resident	0.63	0.31	0.03	1.26	1.89	0.042*
Night worker	0.19	0.18	−0.14	0.54	1.23	0.26
Alcohol consumption	0.38	0.2	−0.01	0.77	1.47	0.057
Use of psychotropic drugs	1.46	0.64	0.24	2.83	4.32	0.023*
Psychotherapy	1.06	0.42	0.23	1.9	2.89	0.012*

For each variable, the table reports the estimated coefficient (log-odds), the standard error (Std. Error), the 95% confidence interval (ci.lower, ci.upper), the corresponding odds ratio (OR), and the p-value testing the null hypothesis of no association. Statistically significant associations are highlighted as follows: ^**^p < 0.01, ^*^p < 0.05, ^.^*p* < 0.1.

### Physical violence

A total of 25 HCWs (9%) stated that they had experienced physical violence at work.

Regarding gender differences, the results showed that 17 (68%) males experienced physical violence (*p* < 0.001).

About age differences, the group of HCWs most affected by physical violence was the 30–39 age group with 11 victims (44%), followed by the 20–29 (*n* = 7, 28%) and 50–59 (*n* = 7, 28%) years age groups.

Referring to the job role, it emerges that the group of nurses was the most affected with 10 (40%) victims, followed by medical doctors with 8 (32%) subjects, 5 (20%) resident doctors and 2 (8%) healthcare auxiliary, reporting having suffered an episode of physical violence. About night work, of the 25 total victims, 18 (72%) HCWs worked at night regularly.

The results referring to years of employment showed that subjects working < 4 years (*n* = 18, 72%) were the HCWs that have suffered the most episodes of this type of violence.

It emerged that 16 (64%) HCWs had suffered violence from a patient, 7 (28%) from a member of staff and 2 (8%) from a manager (*p* < 0.001).

To the question *“How did you react to the physical violence?*,” the most common answer (*n* = 10, 40%) was *“I did not take any action*,” followed by the answers *“I tried to pretend it never happened”* (*n* = 5), *“I reported it to a senior member of staff”* (*n* = 5) and *“I told the person to stop”* (*n* = 5) (*p* = 0.002).

Another question investigated in the case of physical violence was that concerning the symptoms suffered following the event. The most frequent answers were *“Being super-vigilant or on guard”* in 60% (*n* = 15) of the cases and *“Avoiding thinking or talking about the attack, or avoiding having feelings related to it”* in 40% (*n* = 10) of the cases (*p* > 0.05).

It was then asked whether any action had been taken to investigate the causes of the violence: 16 subjects (64%) stated that no action had been taken to investigate the causes, 7 subjects (28.0%) said yes, and 2 (8.0%) did not know whether causes had been investigated or not (*p* < 0.001).

Finally, the last question investigated what the consequences for the aggressor were. The common answers were: “No consequences” (50%) and “Reported to the police” (50%).

### Verbal violence

A total of 227 (85%) HCWS stated that they had experienced verbal violence in the workplace (*p* < 0.001).

128 (56%) were women, 99 (44%) men. About age groups, the most affected age group was 30–39 years old with 94 (41%) HCWs, followed by 67 (30%) HCWs in the 20–29 years old group and 50–59 (*n* = 42, 18%) years age groups.

Regarding the job role, the most affected group was nurses with 72 (32%) cases, followed by medical doctors with 61 (27%) and resident doctors 55 (25%). With regard to the variable of night work 165 (73%) performed night work.

About years of work service, it emerges that subjects working from <4 years are the most affected group, in 50% (*n* = 113) of cases.

The 227 (100%) HCWs who had experienced verbal violence answered to the question as to how often they had experienced this form of violence as followed: 73 (32%) HCWs had experienced verbal violence once, 136 (60%) sometimes and 18 subjects (8%) had experienced it all the time.

About the perpetrators of verbal violence, the results showed that the groups that turned out to be the greatest perpetrators of this form of violence were patients, in 50% of cases, followed by patients’ relatives in 29% and managers/supervisors in 21% of cases (*p* < 0.001).

Regarding the issue of reporting, 161 (71%) subjects reported that they did not report. In particular, 117 (73%) stated that they had not done so because it would have been pointless (*p* < 0.001).

The answers to the question investigating reactions to verbal violence and, among the possible answers, the most frequent response was “I did not take any action,” in 39% of cases (*p* < 0.001).

When asked “Have any actions been taken to investigate the causes of verbal violence?,” 195 (86%) said no, 15 (6%) said they did not know, and only 18 (8%) subjects said yes (*p* < 0.001).

About consequences for the aggressor, the most frequent answer was “Verbal warning issued” in 52% of the cases (*p* > 0.05).

### Bullying

A total of 69 (26%) HCWs stated that they had been bullied in their workplace (*p* < 0.001).

About gender differences, it emerged that more women (*n* = 49, 71%) than men (*n* = 30, 29%) suffered this form of violence.

Regarding the age variable, it emerged that the group most affected by this form of violence was the 20–29 age group (33%), followed by the 30–39 age group (20%), 50–59 age group (38%) age groups and 60–69 age group (9%).

Regarding work role, the group most affected was that of nurses (28%), followed by resident doctors (27%) and then medical doctors (21%).

About night work, on the other hand, 48 (70%) victims of bullying perform night work regularly.

As regards the years of work service, the majority of the victims 33 (48%) belong to the group of subjects who have been working for <4 years, followed by subjects (*n* = 23, 33%) who had been working for more than 10 years and 13 (19%) HCWs who had been working for more than 20 years.

The bullied subjects answered the question as to how often they had experienced this form of violence: 42 (61%) subjects said they had been bullied sometimes, while 21 (30%) said they had been bullied once and 6 (9%) all the time.

Regarding aggressors, most of them (37%) were bullied by the manager/supervisor (*p* > 0.05) and 33% were bullied by a colleague (*p* = 0.013).

Concerning the issue of reporting, 55 (80%) subjects answered this question and stated that they had not reported the incident, because they did not report it because they considered it useless (*p* > 0.05).

The answers to the question concerning how they had reacted to the event: most of the subjects (47%) stated that they had not taken any action (*p* > 0.05).

The next question asked whether any action had been taken to investigate the causes of the bullying suffered: 46 (67%) said no (*p* < 0.001).

Finally, regarding the consequences for the aggressor: 40% stated that they did not know what the consequences were for the aggressor, while 30% stated that there was no consequence and the remaining 30% that the consequence was a verbal warning (*p* > 0.05).

### Sexual harassment

14 (5%) subjects of the total sample confessed to having experienced sexual harassment in the workplace (*p* < 0.001).

Regarding gender differences, of the 14 subjects who experienced sexual harassment, 11 (79%) were women.

With respect to age, 8 (57%) subjects of the victims belong to the 30–39 age group, 5 (36%) to the 20–29 age group and 1 (7%) to the 40–49 age group.

With reference to the job role variable, it emerged that the group of resident doctors was the most affected (54%), followed by nurses (31%). For the variable of night work, 11 (79%) HCWs performed night work regularly.

About the variable of years of service, the majority of the victims (79%) belonged to the group of subjects working < 4 years.

The 14 (100%) subjects who experienced sexual harassment answered to the question as to how often they had experienced this form of violence. 8 (60%) subjects stated that they had experienced sexual harassment a few times, while 6 (40%) had experienced it once.

To the question “Who sexually harassed you?,” the majority (48%) stated that they had experienced this form of violence from a patient (*p* > 0.05).

About the issue of reporting, 50% HCWs stated that they did not report the incident because they considered it useless (*p* > 0.05).

As regards the victims’ reactions to sexual harassment, the most frequent answers (21%) were: “I did not take any action,” “I reported it to a senior member of staff” and “I told a colleague” (*p* > 0.05).

The next question asked whether any action had been taken to investigate the causes of the sexual harassment experienced: 13 (93%) HCWs stated that no action had been taken to investigate the causes of the incident (*p* = 0.003). The only subject who answered in the affirmative then stated that there were no consequences for the aggressor.

### Racial harassment

5 (2%) HCWS of the total sample reported having experienced racial harassment (*p* < 0.001). Racial harassment, therefore, is the less frequent form of violence in the sample of this study.

As regards gender differences, it was more the males (80%) who were racially harassed.

Compared to age, it appears that the groups most affected (40%) were those in the 20–29 and 30–39 years old age groups.

In terms of the occupational role variable, the group most affected (40%) was that of nurses. As regards night work, 4 subjects (80%) worked regularly at night.

As regards the years of work service, most victims (60%) work from <4 years, while the other 40% victims belong to the groups of working persons from 10 to 14 years.

To the question asking how many times they had been racially harassed: 3 subjects (60%) stated that they had suffered this form of violence “sometimes,” while 2 (40%) had experienced it “once.”

3 (60%) had experienced racial harassment by a patient’s relatives, 1 (20%) by an executive/supervisor and 1 (20%) by a staff member (*p* > 0.05).

As regards the complaint problem, all respondents to this question stated that they did not file a complaint because they considered it unnecessary (*p* > 0.05).

The answers to the question about their reactions to racial harassment were: “I did not take any action” (2); “I tried to pretend that it never happened” (1); “I reported it to a senior staff member” (1); “I asked for help from the unions” (1) (*p* > 0.05).

Finally, all five victims admitted that no action was taken to investigate the causes of racial harassment.

## Discussion

Violence against healthcare workers is a growing problem that manifests itself in many forms, from physical assaults to verbal threats. It is a phenomenon that endangers the safety of staff and compromises the quality of patient care.

The findings from this study provide significant insights into the prevalence and nature of WPV against HCWs in Southern Italy. Our results are largely consistent with international and national trends but also highlight specific contextual factors that warrant attention.

It should be noted that, since the questionnaire specifically referred to events that occurred in the last 12 months, we excluded staff with shorter tenures to avoid introducing bias due to partial exposure time.

As detected by Liu et al. (14), throughout their working lives about 60% of HCWs experienced some form of violence, with 24.4% subjected to physical violence. Also, our study found that 27% (*n* = 267) of HCWs reported experiencing violence in the past year, aligning with these global figures.

The group of nurses seems to be the most affected by violence, 30% of the sample. Similar results were found in a recent study by Doehring et al. (20) who found a higher incidence of cases of violence in nurses, especially in those under 40 years of age, among HCWs. The episodes of violence were observed with a frequency of 1 case every 3.7 work shifts (20), with no differences between night and day shifts.

Similarly, in the present study 65% of HCWs performed shift work including night shift regularly; but in line with literature data, no episodes of violence related to the night shift or to a specific shift were recorded.

A possible explanation for the greater involvement of nurses compared to other HCWs could be linked to the particular role they play, who spend many hours of care with patients and come into greater contact with the relatives of the same patients (21).

Often, nurses are the patient’s first interlocutors who can address his feelings of anger toward them (22).

The prevalence of verbal violence, 85% in the present study, is similar to that found in several other studies; in particular, verbal abuse is more common than physical violence (22). However, prevalence rates of WPV vary significantly between Countries: Australia and North America report the highest incidence rates of all forms of violence in HCWs, 70.9 and 67.3%, respectively; while Europe records the lowest rate, 48.1% (23). The results of the present study are consistent with European trends but slightly higher, perhaps reflecting regional specificities in Southern Italy. Because violence is a phenomenon influenced by culture and context, the prevalence of WPV varies from region to region (24).

In 2022, a cross-sectional study conducted on HCWs operating in several Italian Regions (15) with the aim of investigating the prevalence of some forms of WPV showed that a significant difference was found between the different regions, such that those in the North had a higher incidence of bullying (17%). La Torre et al. (15) identified higher incidences of bullying and harassment among nurses and women, a trend also observed in this study where nurses and female HCWs were more frequently victims of violence. However, our study indicated a younger age group (20–29 years) as more affected by bullying, contrasting with La Torre et al. (15) findings which highlighted the 35–39 age group. This difference could be attributed to recent staff hires related to turnover.

Furthermore, our results align with national data indicating that sexual harassment strongly affects female HCWs, although our findings suggest a higher prevalence in the 30–39 age group compared to the 20–24 age group reported by La Torre et al. (15).

Additionally, the prevalence of physical violence in our study (9%) is lower than the global average but remains a critical concern, particularly given the higher susceptibility of psychiatric and emergency department staff as noted globally (22). When comparing our findings with national data from Italy, similar patterns emerge (15).

A recurrent theme in both our study and the broader literature is the underreporting of WPV (25). The majority of HCWs in our study did not report incidents of violence, primarily because they perceived reporting as futile or feared negative repercussions. This aligns with data reported by Rossi et al. and Alsharari et al. (17, 18), which underscore the normalization of violence in healthcare workplace and the lack of trust in reporting mechanisms. The absence of effective reporting systems, as evidenced by Arnetz et al. (26), exacerbates the issue, suggesting a critical need for institutional reforms to encourage reporting and ensure follow-up actions. Furthermore, study highlights specific reasons for underreporting, such as the belief that violence is an inherent part of healthcare work, particularly in high-stress environments like emergency rooms and psychiatric wards. This normalization of violence not only discourages reporting but also perpetuates a cycle of acceptance and exposure. The absence of significant sociodemographic differences between HCWs who experienced violence and those who did not may reflect a widespread exposure to risk factors across professional roles and departments. Alternatively, it could be due to underreporting in certain groups or the homogenizing effect of shared organizational stressors such as workload, shift work, and staffing shortages.

This study highlights the significant psychological burden WPV places on HCWs. Increased use of psychotropic medications and psychotherapy among victims indicates the severe mental health consequences of such violence. This is consistent with literature linking WPV to conditions such as burnout, anxiety, depression, and post-traumatic stress disorder (PTSD) (27, 28). Specific symptoms reported by our participants, such as hyper-vigilance, avoidance behaviors, and intrusive thoughts, align with PTSD criteria, underscoring the urgent need for psychological support services (27, 28). Additionally, the potential bidirectional relationship between pre-existing mental health conditions and vulnerability to WPV needs further exploration. Our data suggest that HCWs with existing mental health issues may be more susceptible to violence, possibly due to impaired coping mechanisms or increased exposure to high-risk situations.

Gender appeared to have a relevant role in the experience of workplace violence, with women more frequently reporting exposure to all types—particularly verbal abuse, bullying, and sexual harassment. Although these differences were not statistically significant, the observed trend aligns with national and international literature suggesting that female HCWs often face higher risks due to workplace dynamics, social norms, and professional hierarchies. Structural barriers such as fear of retaliation, stigma, and normalization of abuse may hinder reporting among women, potentially worsening the psychological consequences of violence. Gender-sensitive prevention and support strategies should be considered to promote safer and more equitable working conditions.

The analysis of the results highlighted the need to intervene to try to prevent the phenomenon of WPV and to support victims regarding the possible psychological consequences that could arise from it. The analysis of the sample showed that almost 5% of workers were taking psychotropic drugs and that almost 12% were being treated by a psychotherapist; this indicates the presence of real pathological states that required specific treatments. In general, as reported in the literature, the main psychological symptoms that could arise following exposure to violence at work are: tiredness, difficulty concentrating and forgetfulness, irritability, low self-esteem, low self-efficacy and demotivation. In fact, these symptoms could also be considered risk factors for greater exposure to violence, because it is known that excessive workload and stressful working conditions in which healthcare workers work daily can lead to the development of these symptoms, linked to burnout syndrome (19). In fact, burnout is a syndrome that arises from chronic work-related stress that can have significant negative consequences on a person’s health such as severe stress, which is both a cause and a consequence; tiredness and weakness, including mental; sleep and concentration disorders; pain, palpitations and dizziness; loss of motivation and intense tension; low self-esteem and emotional instability (7).

Regarding the stress that victims of violence can experience at work, one of the main psychological consequences is also Post-Traumatic Stress Disorder, as can be seen especially in the answers that the subjects gave to the question “Following the physical violence event you suffered:” where the main answers were: “Be super-vigilant or on guard,” “Avoid thinking or talking about the attack or avoid having symptoms related to it” and “Thoughts or images of the attack” (29, 30).

In light of the findings, it is critical to implement comprehensive interventions at both the organizational and individual levels. Organizational strategies should include enhanced reporting mechanisms, training and education on WPV prevention, and the establishment of support systems such as psychological counseling and peer support groups. Policy reforms are also needed to enforce zero-tolerance policies against WPV and protect health workers from retaliation. At the individual level, psychological first aid and long-term therapy, including innovative approaches such as hypnosis combined with virtual reality, may be effective in mitigating the psychological impact of WPV.

Paradoxically, the rate of physical attacks diminished during the pandemic when the perpetrators were almost exclusively patients who were not in full possession of their mental faculties (25), most likely due to the filtering of access to work areas that had limited the presence of visitors and relatives. Field studies demonstrate that unrestricted access to working areas, the lack of security guards and police officers, and the limited intervention on their part are among the causes of violence (31). Only an integrated strategy on several levels is likely to reduce violence in the HCW’S.

It would be good practice to have an annual meeting with workers, employers and other figures involved in health and safety at work in order to report any assaults so that adequate prevention plans can be developed.

Legislative Decree no. 137/2024 “Urgent measures to combat violence against healthcare, social healthcare, auxiliary and assistance and care professionals in the exercise of their functions as well as damage to goods intended for healthcare” introduces specific measures to combat violence in the workplace, recognizing the need to protect healthcare personnel more effectively. The decree takes into account the growing reports of episodes of physical and verbal aggression against doctors, nurses and other healthcare workers.

The limitations of this study are due to the use of self-reported data, in fact underreporting remains a concern when using self-reported data, particularly in organizational cultures where violence may be normalized or feared to be reported. This methodological limitation has been widely recognized in WPV literature. Furthermore, the study was conducted in a single center located in a large city in Southern Italy. Future research should be multicenter and incorporate longitudinal designs and expand to healthcare settings to validate and extend these findings. While our study acknowledges the limitations inherent in a cross-sectional design, it is important to note that the associations observed do not imply causation. Particularly in the discussions surrounding PTSD and drug use, we recognize that these variables may be bidirectionally related, and other confounding factors could be at play. Future research employing longitudinal designs is necessary to better elucidate these complex relationships.

## Conclusion

In conclusion, this study confirms that WPV is a widespread and multifaceted problem among HCWs in Southern Italy, with significant psychological repercussions that reflect global and national trends. Our study highlights the prevalence of verbal abuse and the significant psychological toll on victims, highlighting the urgent need for comprehensive reporting mechanisms and psychological support. The normalization of violence in healthcare settings remains a critical obstacle to addressing this problem, requiring targeted interventions to change cultural perceptions and improve worker safety. An important role can be played by the occupational physician who, during the periodic medical examination, can intercept early-onset psychological disorders and/or emotional alterations and initiate the worker to specific psychological support pathways. It is important to address the problem of WPV by coordinating efforts at both organizational and political levels to promote a safer and more supportive work environment for HCWs.

## Data Availability

The raw data will be made available by the authors without undue reservation.

## References

[1] Rodwell J Demir D. Oppression and exposure as differentiating predictors of types of workplace violence for nurses. J Clin Nurs. (2012) 21:2296–305. doi: 10.1111/j.1365-2702.2012.04192.x, PMID: 22788563

[2] Wiskow C. Joint programme on workplace violence in the health sector. Geneva: workplace violence in the health sector country case studies research instruments. Survey questionnaire. (2003). WHO.

[3] Ramaci T Barattucci M Vella F Senia P Cannizzaro E Scorciapino A. Straining at work and its relationship with personality profiles and individual consequences in healthcare workers (HCWs). Int J Environ Res Public Health. (2020) 17:610. doi: 10.3390/ijerph17020610, PMID: 31963612 PMC7027001

[4] Moro MF Calamandrei G Poli R Di Mattei V Perra A Kurotschka PK. The impact of the COVID-19 pandemic on the mental health of healthcare Workers in Italy: analyzing the role of individual and workplace-level factors in the reopening phase after lockdown. Front Psych. (2022) 13:867080. doi: 10.3389/fpsyt.2022.867080, PMID: 35722544 PMC9200968

[5] Ferri P Silvestri M Artoni C Di Lorenzo R. Workplace violence in different settings and among various health professionals in an Italian general hospital: a cross-sectional study. Psychol Res Behav Manag. (2016) 9:263–75. doi: 10.2147/PRBM.S114870, PMID: 27729818 PMC5042196

[6] Ramaci T Pellerone M Ledda C Rapisarda V. Health promotion, psychological distress, and disease prevention in the workplace: a cross-sectional study of Italian adults. Risk Manag Healthc Policy. (2017) 10:167–75. doi: 10.2147/RMHP.S139756, PMID: 28860882 PMC5565380

[7] Tsukamoto SAS Galdino MJQ Barreto MFC Martins JT. Burnout syndrome and workplace violence among nursing staff: a cross-sectional study. Sao Paulo Med J. (2021) 140:101–7. doi: 10.1590/1516-3180.2021.0068.R1.31052021 PMC9623842 34932780

[8] Muratori F Frenos F Bettini L Capanna R Campanacci DA. Liposarcoma: Clinico-pathological analysis, prognostic factors and survival in a series of 307 patients treated at a single institution. J Orthopaed Sci. (2018) 23:1038–44. doi: 10.1016/j.jos.2018.06.008, PMID: 30007495

[9] Balducci C Vignoli M Dalla Rosa G Consiglio C. High strain and low social support at work as risk factors for being the target of third-party workplace violence among healthcare sector workers. Med Lav. (2020) 111:388–98. doi: 10.23749/mdl.v111i5.9910, PMID: 33124610 PMC7809982

[10] Ramacciati N Gili A Mezzetti A Ceccagnoli A Addey B Rasero L. Violence towards emergency nurses: the 2016 Italian national survey—a cross-sectional study. J Nurs Manag. (2019) 27:792–805. doi: 10.1111/jonm.12733, PMID: 30430675

[11] Wu J Tung T Chen PY Chen Y Lin Y Chen F. Determinants of workplace violence against clinical physicians in hospitals. J Occup Health. (2015) 57:540–7. doi: 10.1539/joh.15-0111-OA, PMID: 26423827 PMC6706174

[12] Kumari A Kaur T Ranjan P Chopra S Sarkar S Baitha U. Workplace violence against doctors: characteristics, risk factors, and mitigation strategies. J Postgrad Med. (2020) 66:149–54. doi: 10.4103/jpgm.JPGM_96_20, PMID: 32675451 PMC7542052

[13] Kuehn BM. Violence in health care settings on rise. JAMA. (2010) 304:511–2. doi: 10.1001/jama.2010.1010, PMID: 20682926

[14] Liu J Gan Y Jiang H Li L Dwyer R Lu K. Prevalence of workplace violence against healthcare workers: a systematic review and meta-analysis. Occup Environ Med. (2019) 76:927–37. doi: 10.1136/oemed-2019-105849, PMID: 31611310

[15] La Torre G Firenze A Colaprico C Ricci E Di Gioia LP Serò D. Prevalence and risk factors of bullying and sexual and racial harassment in healthcare workers: a cross-sectional study in Italy. Int J Environ Res Public Health. (2022) 19:6938. doi: 10.3390/ijerph19116938, PMID: 35682522 PMC9180018

[16] D’Alterio N Fantinelli S Galanti T Cortini M. The mediator role of the job related stress in the relation between learning climate and job performance. Evidences from the health sector. Recenti Prog Med. (2019) 110:251–4. doi: 10.1701/3163.31448 31140458

[17] Rossi MF Beccia F Cittadini F Amantea C Aulino G Santoro PE. Workplace violence against healthcare workers: an umbrella review of systematic reviews and meta-analyses. Public Health. (2023) 221:50–9. doi: 10.1016/j.puhe.2023.05.021, PMID: 37406450

[18] Alsharari AF Abu-Snieneh HM Abuadas FH Elsabagh NE Althobaity A Alshammari FF. Workplace violence towards emergency nurses: a cross-sectional multicenter study. Australas Emerg Care. (2022) 25:48–54. doi: 10.1016/j.auec.2021.01.004, PMID: 33602656

[19] Aristidou L Mpouzika M Papathanassoglou EDE Middleton N Karanikola MNK. Association between workplace bullying occurrence and trauma symptoms among healthcare professionals in Cyprus. Front Psychol. (2020) 11:575623. doi: 10.3389/fpsyg.2020.575623, PMID: 33281676 PMC7688662

[20] Doehring MC Palmer M Satorius A Vaughn T Mulat B Beckman A. Workplace violence in a large Urban emergency department. JAMA Netw Open. (2024) 7:e2443160. doi: 10.1001/jamanetworkopen.2024.43160, PMID: 39499514 PMC11539014

[21] Cedeno R Bohlen J. Sexual harassment and prevention training In: StatPearls. Treasure Island (FL): StatPearls Publishing (2024) 36508513

[22] Doehring MC Curtice H Hunter BR Oaxaca DM Satorius A Reed K. Exploring verbal and physical workplace violence in a large, urban emergency department. Am J Emerg Med. (2023) 67:1–4. doi: 10.1016/j.ajem.2023.01.036, PMID: 36758267

[23] Reader TW Gillespie A. Patient neglect in healthcare institutions: a systematic review and conceptual model. BMC Health Serv Res. (2013) 13:156. doi: 10.1186/1472-6963-13-156, PMID: 23631468 PMC3660245

[24] Ramzi ZS Fatah PW Dalvandi A. Prevalence of workplace violence against healthcare workers during the COVID-19 pandemic: a systematic review and Meta-analysis. Front Psychol. (2022) 13:896156. doi: 10.3389/fpsyg.2022.896156, PMID: 35712196 PMC9195416

[25] Magnavita N Meraglia I Viti G Gasbarri M. Tracking workplace violence over 20 years. Int J Environ Res Public Health. (2024) 21:1438. doi: 10.3390/ijerph21111438, PMID: 39595705 PMC11593827

[26] Arnetz JE Hamblin L Ager J Luborsky M Upfal MJ Russell J. Underreporting of workplace violence. Workplace Health Saf. (2015) 63:200–10. doi: 10.1177/2165079915574684, PMID: 26002854 PMC5006066

[27] D'Ettorre G Pellicani V Ceccarelli G. Post-traumatic stress disorder symptoms in healthcare workers: a ten-year systematic review. Acta Biomed. (2020) 91:e2020009. doi: 10.23750/abm.v91i12-S.9459, PMID: 33263341 PMC8023102

[28] Nam SH Lee DW Seo HY Hong YC Yun JY Cho SJ. Empathy with patients and post-traumatic stress response in verbally abused healthcare workers. Psychiatry Investig. (2021) 18:770–8. doi: 10.30773/pi.2021.0066, PMID: 34404121 PMC8390940

[29] Sasson Y Dekel S Nacasch N Chopra M Zinger Y Amital D. Posttraumatic obsessive-compulsive disorder: a case series. Psychiatry Res. (2005) 135:145–52. doi: 10.1016/j.psychres.2004.05.026, PMID: 15922457

[30] Cloitre M Koenen KC Cohen LR Han H. Skills training in affective and interpersonal regulation followed by exposure: a phase-based treatment for PTSD related to childhood abuse. J Consult Clin Psychol. (2002) 70:1067–74. doi: 10.1037//0022-006x.70.5.1067, PMID: 12362957

[31] Antão HS Sacadura-Leite E Manzano MJ Pinote S Relvas R Serranheira F. Workplace violence in healthcare: a single-center study on causes, consequences and prevention strategies. Acta Medica Port. (2020) 33:31–7. doi: 10.20344/amp.11465, PMID: 31928601

